# Correction: Application of LDA and word2vec to detect English off-topic composition

**DOI:** 10.1371/journal.pone.0312710

**Published:** 2024-10-21

**Authors:** The PLOS ONE Editors

## Notice of Republication

At the time of retraction, the article [1] remained available online. The retracted article was subsequently removed from the *PLOS ONE* website on October 1, 2024 and further updates were made to the Copyright statement and the Data Availability statement. The Retraction [2] was republished on October 2, 2024 to include an update notifying readers of the removal of the article content.
